# Zoster sine herpete following BNT162b2 mRNA COVID-19 vaccination in an immunocompetent patient

**DOI:** 10.1016/j.idcr.2022.e01563

**Published:** 2022-07-13

**Authors:** Ryutaro Tanizaki, Yayoi Miyamatsu

**Affiliations:** Department of Internal Medicine and General Medicine, Ise Municipal General Hospital, 3038, Kusubecho, Ise, Mie 516-0014, Japan

**Keywords:** Zoster sine herpete, COVID-19, Varicella zoster virus, mRNA vaccination

## Abstract

As a result of the COVID-19 pandemic, mRNA vaccination has become widespread. Recently, it has been suggested that instances of herpes zoster increase following mRNA COVID-19 vaccination. Herein, we describe the first case of zoster sine herpete (ZSH) after mRNA vaccination for COVID-19. A 60-year-old Japanese immunocompetent man presented with fever, fatigue, headache, cervical pain, and lumbar pain, which developed after receiving a second dose of BNT162b2 mRNA COVID-19 vaccination. Whereas most symptoms improved with symptomatic treatment, headache and numbness of the right forehead persisted in areas innervated by the trigeminal and second and third cervical nerves. Based on positive results of an enzyme-linked immunosorbent assay examination for anti-VZV IgM, ZSH was diagnosed, and amitriptyline improved the patient’s symptoms. Diagnosis of ZSH is challenging due to the lack of a characteristic herpes zoster rash. Physicians should be aware that ZSH can develop after mRNA vaccination.

## Introduction

During the COVID-19 pandemic, mRNA vaccination has been conducted worldwide, which has demonstrated a high efficacy rate with an acceptable safety profile [1]. Recently, it has been suggested that herpes zoster potentially increases following mRNA COVID-19 vaccination [2], [3], [4] although it is uncertain if mRNA COVID-19 vaccine significantly induced that [5].

Herpes zoster, which is caused by the reactivation of varicella-zoster virus (VZV), especially in an immunocompromised host, typically presents a painful rash along with a suffered innervation area. Rarely, there is an atypical type of zoster called zoster sine herpete (ZSH) that is lack the characteristic rash despite the presence of typical dermatomal pain due to VZV [6]. Several reports regarding herpes zoster following mRNA COVID-19 vaccination have been described, no cases of ZSH have been reported. Herein, we described the first case of ZSH after the mRNA vaccination of COVID-19.

## Case report

In December 2021, a 60-year-old Japanese immunocompetent man with type-2 diabetes mellitus presented to the emergency department (ED) with fever, headache, sore throat, and lumbar pain. He had been taking vildagliptin metformin hydrochloride and luseogliflozin hydrate. Whereas he did not receive vaccination of herpes zoster, he received the second dose of BNT162b2 mRNA COVID-19 vaccination 10 days before ED visit. The next day of vaccination, fatigue, lumbar pain, and harsh headache appeared, and subsequently, a fever of 38 °C and sore throat developed more 2 days after. Seven days after vaccination numbness and tingling skin pain developed on the right area of the forehead. A head computer tomography conducted at the neurosurgeon clinic showed normal. Ten days after vaccination, he presented to ED in our hospital. He complained that the headache was the worst among his symptoms, and the pain involved the entire head tightening and mainly occurred from the frontal region to the temporal region on the right-side. The intermittent pain momentarily strikes, and even just touching the hair hurt.

On physical examination, a body temperature of 36.4 °C and other vital signs were normal. No physical abnormalities were observed including his skin. Nuchal rigidity and jolt accentuation were negative. His laboratory tests revealed within normal except slightly elevated C-reactive protein (0.27 mg/dl [reference range: 0–0.14 mg/dl]).

Oral acetaminophen was administered, and he went back home. Two days after the ED visit, he was introduced to the department of General Medicine. He was afebrile and generally well, all symptoms were recovered except headache and numbness of right forehead. His fatigue, lumbar pain, and sore throat were almost improved. Based on the nature of his symptoms, he was initially considered the side effects of mRNA vaccination complicated with an incidentally developed trigeminal neuralgia, resulting in the administration of 400 mg/day of carbamazepine. Two weeks after starting carbamazepine, his symptoms slightly improved, but strong pain remained. Therefore, 10 mg of amitriptyline was administered instead of carbamazepine by presuming the pain due to ZSH. Then, his symptoms dramatically improved within a few days of taking amitriptyline. Thereafter, it was found that an enzyme-linked immunosorbent assay (ELISA) examination of anti-VZV IgM antibody was positive (also anti-VZV IgG was positive) measured 26 days after the most recent mRNA vaccination.

Finally, neuralgia due to ZSH was diagnosed, and no symptoms were observed at the outpatient visit 2 weeks after amitriptyline was administered. No recurrence of symptoms has been observed so far.

## Discussion

Many cases of herpes zoster following COVID-19 vaccination were reported [3], [7], to our knowledge, this case is the first case of ZSH following mRNA vaccination. Among herpes zoster cases following COVID-19 vaccination, several cases have been reported after mRNA vaccination [2], [3], [4]. However, it might simply be due to the high frequency of mRNA vaccination worldwide. Birabaharan et al. reported that no difference in VZV reactivation was observed among persons receiving the mRNA COVID-19 vaccine compared to the comparison group in their cohort study with propensity score matching [5].

ZSH presents a variety of syndromes such as unilateral dermatomal pain, muscular paresis, segmental pain associated with certain visceral disturbances, ophthalmic neuralgia, otalgia, labyrinthitis, and paralysis of the soft palate, pharyngeal muscles, or vocal code [10]. Thus, without skin lesion, it may be difficult to remind of herpes zoster in patients with the above symptoms; namely, the diagnosis of ZSH with symptoms alone can lead to misdiagnosis. In this case, clinicians initially assumed that all the patient’s symptoms were caused by side effects of the vaccination, which might have resulted in the diagnostic delay and miss the opportunity of timely antiherpetic agents administration. Indeed fever, lumbar pain, and headache were reasonable symptoms for side effects of the vaccine, but it should have been more cautious that sore throat and numbness of limited right forehead were uncommon symptoms of that [8].

For the accurate diagnosis of ZSH, VZV DNA with polymerase chain reaction (PCR) in saliva and anti-VZV IgM and IgG antibodies in serum and cerebrospinal fluid (CSF) are available. Especially, anti-VZV IgG antibody in CSF may be more useful [9] regardless of the presence of meningitis, but it is not realistic to perform a lumbar puncture on all the patients suspected of ZSH especially in the primary care setting. Despite the risk of false-negative results in an early phase of disease and being time-consuming, anti-VZV IgM antibody is considered useful for the diagnosis [10]. In this patient, although lumbar puncture was not performed, ZSH was diagnosed based on the positive result of the anti-VZV IgM antibody. PCR was not performed because it is insured only for immunocompromised patients in Japan.

**Fig. 1:**
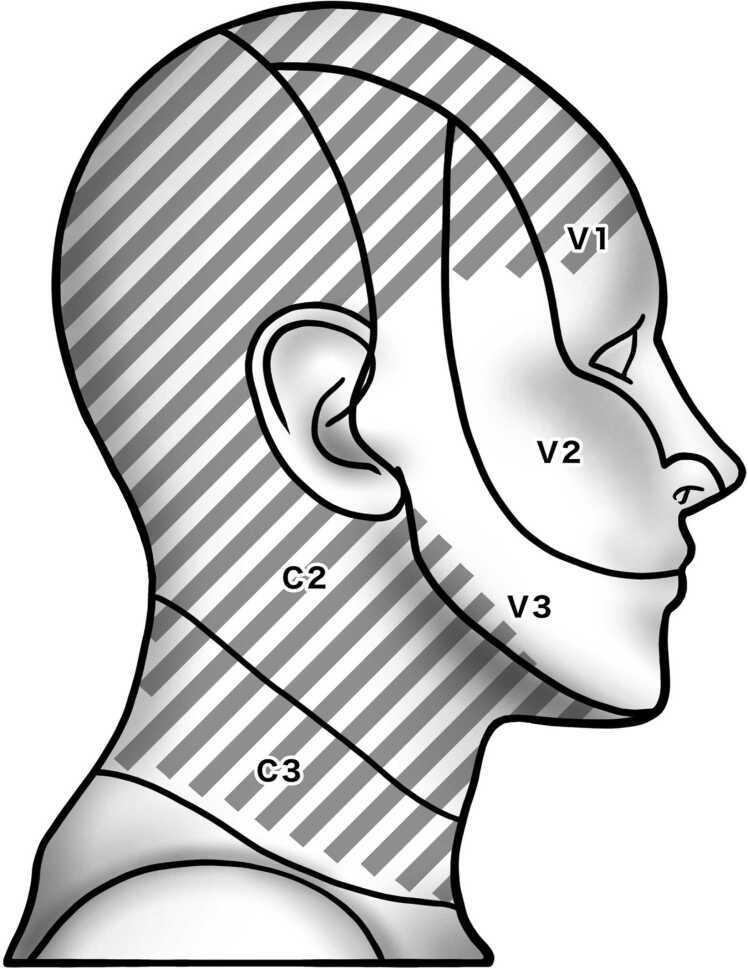
The area of patient’s symptoms. The shaded area is where he complained of pain. He reported that the pain spread from the right frontal region to the right temporal and occipital regions. It then spread from the right cervical region to the right anterior cervical region and radiated towards the right shoulder.

In this patient, an antiviral drug for herpes zoster was not administered because it had been 3 weeks after the symptoms’ onset. Furthermore, this patient’s pain developed even mere touching the hair, considering allodynia, which indicated a prominent symptom of postherpetic neuralgia (PHN). Based on these symptoms, the treatment for neuropathic pain was considered more indispensable than that for the reactivation of VZV. Although carbamazepine was initially administered to the patient, it was less effective. Carbamazepine is not necessarily recommended for the treatment of PHN [11], and as oral medication, gabapentin, pregabalin, tricyclic antidepressants, and opioids are suggested [11]. In this case, amitriptyline improved the patient’s symptoms (Fig. 1).

ZSH can develop following mRNA vaccination. The diagnosis of ZSH is challenging due to lack of characteristic rash as herpes zoster regardless of COVID-19 vaccination. Because early detection and treatment for herpes zoster are essential to shorten the length and severity of the illness, in patients with this pain caused by obscure origin, clinicians should be aware of ZSH as a differential diagnosis even though the skin appearance is intact.

## CRediT authorship contribution statement

All authors meet the ICMJE authorship criteria. The manuscript was written by RT and YM. The authors approved the final manuscript.

## ICMJE statement

All authors meet the ICMJE authorship criteria.

## Funding

This research did not receive any specific grant from funding agencies in the public, commercial, or not-for-profit sectors.

## Ethical approval

Not applicable.

## Consent

Written informed consent was obtained from the patients.

## Conflict of Interest

The authors have declared no conflict of interests.
